# Development of an EGFRvIII specific recombinant antibody

**DOI:** 10.1186/1472-6750-10-72

**Published:** 2010-10-07

**Authors:** Puja Gupta, Shuang-Yin Han, Marina Holgado-Madruga, Siddhartha S Mitra, Gordon Li, Ryan T Nitta, Albert J Wong

**Affiliations:** 1 Brain Tumor Research Laboratories, Department of Neurosurgery, and Program in Cancer Biology, Stanford University Medical Center, Stanford, CA94305, USA; 2 Department of Gastroenterology, Henan Provincial People's Hospital, Zhenghou, Henen 450003, P.R. China

## Abstract

**Background:**

EGF receptor variant III (EGFRvIII) is the most common variant of the EGF receptor observed in human tumors. It results from the in frame deletion of exons 2-7 and the generation of a novel glycine residue at the junction of exons 1 and 8. This novel juxtaposition of amino acids within the extra-cellular domain of the EGF receptor creates a tumor specific and immunogenic epitope. EGFRvIII expression has been seen in many tumor types including glioblastoma multiforme (GBM), breast adenocarcinoma, non-small cell lung carcinoma, ovarian adenocarcinoma and prostate cancer, but has been rarely observed in normal tissue. Because this variant is tumor specific and highly immunogenic, it can be used for both a diagnostic marker as well as a target for immunotherapy. Unfortunately many of the monoclonal and polyclonal antibodies directed against EGFRvIII have cross reactivity to wild type EGFR or other non-specific proteins. Furthermore, a monoclonal antibody to EGFRvIII is not readily available to the scientific community.

**Results:**

In this study, we have developed a recombinant antibody that is specific for EGFRvIII, has little cross reactivity for the wild type receptor, and which can be easily produced. We initially designed a recombinant antibody with two anti-EGFRvIII single chain Fv's linked together and a human IgG1 Fc component. To enhance the specificity of this antibody for EGFRvIII, we mutated tyrosine H59 of the CDRH2 domain and tyrosine H105 of the CDRH3 domain to phenylalanine for both the anti-EGFRvIII sequence inserts. This mutated recombinant antibody, called RAb^DMvIII^, specifically detects EGFRvIII expression in EGFRvIII expressing cell lines as well as in EGFRvIII expressing GBM primary tissue by western blot, immunohistochemistry (IHC) and immunofluorescence (IF) and FACS analysis. It does not recognize wild type EGFR in any of these assays. The affinity of this antibody for EGFRvIII peptide is 1.7 × 10^7 ^M^-1 ^as determined by enzyme-linked immunosorbent assay (ELISA).

**Conclusion:**

This recombinant antibody thus holds great potential to be used as a research reagent and diagnostic tool in research laboratories and clinics because of its high quality, easy viability and unique versatility. This antibody is also a strong candidate to be investigated for further in vivo therapeutic studies.

## Background

According to Global Health Council despite advances in the understanding of cancer biology, malignant neoplasms remain the second leading cause of mortality in United States. Novel therapies are needed and over the past two decades new immunotherapy strategies have emerged as a promising approach. The success of these immunotherapeutic strategies is highly dependent on finding an ideal antigen to target. One such target is the EGF receptor which is over or aberrantly expressed in a variety of human cancers [1-12]. However, the fact that the EGF receptor is present on normal tissue could lead to possible deleterious side effects or immune tolerance when treating patients with reagents solely targeting the EGF receptor. A tumor specific antigen would be more ideal. Epidermal growth factor receptor variant III (EGFRvIII) is the most common variant of the EGF receptor and is expressed in a number of solid tumors including glioblastoma multiforme (GBM), breast adenocarcinoma, medulloblastoma and ovarian adenocarcinoma, but has only rarely been observed in normal tissue [13-15]. This variant receptor results from an in-frame deletion of exons 2-7 of the wild type EGF receptor. This causes a deletion of a large portion of the extracellular domain and the generation of a novel glycine at the fusion junction between exon 1 and exon 8 [16-18]. EGFRvIII is ligand independent yet constitutively active and when expressed in cells it leads to unregulated growth, survival, invasion, and angiogenesis [19]. EGFRvIII is an ideal target for immunotherapy because the juxtaposition of exon 1 and 8 sequences plus the novel glycine creates a highly immunogenic tumor specific antigen, and the receptor itself leads to a cancer phenotype due to its constitutive activity [1,20-22].

There are a number of immune approaches that specifically target EGFRvIII. For example, a peptide vaccine targeting this mutant receptor is currently being tested in clinical trials for GBM patients [23,24]. Another approach is the development of antibodies that recognize EGFRvIII which can be used for diagnostic/laboratory purposes or for therapeutic purposes either alone or attached to a cytotoxic or radiolabeled adjunct [25,26]. Many of the monoclonal antibodies that target EGFRvIII, however, have cross reactivity with the wild type EGF receptor or other non-specific proteins [27] or comparatively have low affinity. While polyclonal antibodies are apparently highly sensitive and of high affinity, it is difficult to produce them in mass quantities. An additional complicating factor is that the rights to the antibody for EGFRvIII are patented and have not been made widely available for either scientific or medical use.

To generate a highly specific and cost effective antibody that could be used for scientific and clinical purposes, we developed a recombinant antibody. Recombinant antibody technology has gained popularity in recent years because of its many advantages when compared with the production of monoclonal or polyclonal antibodies. First, no animals are needed and the manufacturing time is relatively short compared with conventional methods. Moreover, the quality and quantity of the final product is superior when compared to conventional antibody production. Recombinant antibodies are formed by combining single-chain Fv antibodies, consisting of V_H _and V_L _chains, by a flexible linker [28]. In this report, we describe how we created a recombinant antibody that specifically recognizes EGFRvIII and demonstrate its specificity by western blot analysis, immunohistochemistry (IHC), immunofluorescence (IF), enzyme-linked immunosorbent assay (ELISA) and flow cytometry analysis (FACS). This easily produced and highly specific recombinant antibody holds great potential to be used as a diagnostic and a therapeutic tool.

## Results

### Western Analysis

**Figure 1:**
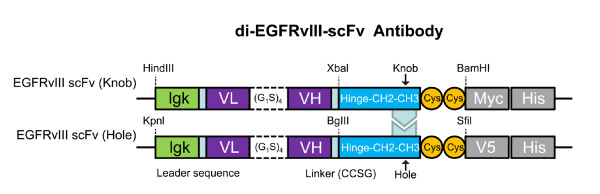
**Schematic of the RAb^DMvIII ^antibody**. The genes for the anti-EGFRvIII scFvs were cloned into the pBudCE4.1 dicistronic vector. The Ig k-chain signal sequence at the 5' end of each sequence allowed the secretion of the molecule in the culture media. "Knobs-into-holes" configuration and the two cysteine residues at the 3' end of each CH3 domain stabilize the construct. Sequence corresponding to a V5 epitope was cloned at the 3' end of one anti-EGFR scFvs and the other anti-EGFR scFv has Myc as well as a 6xHis tag at 3'end.

**Figure 2:**
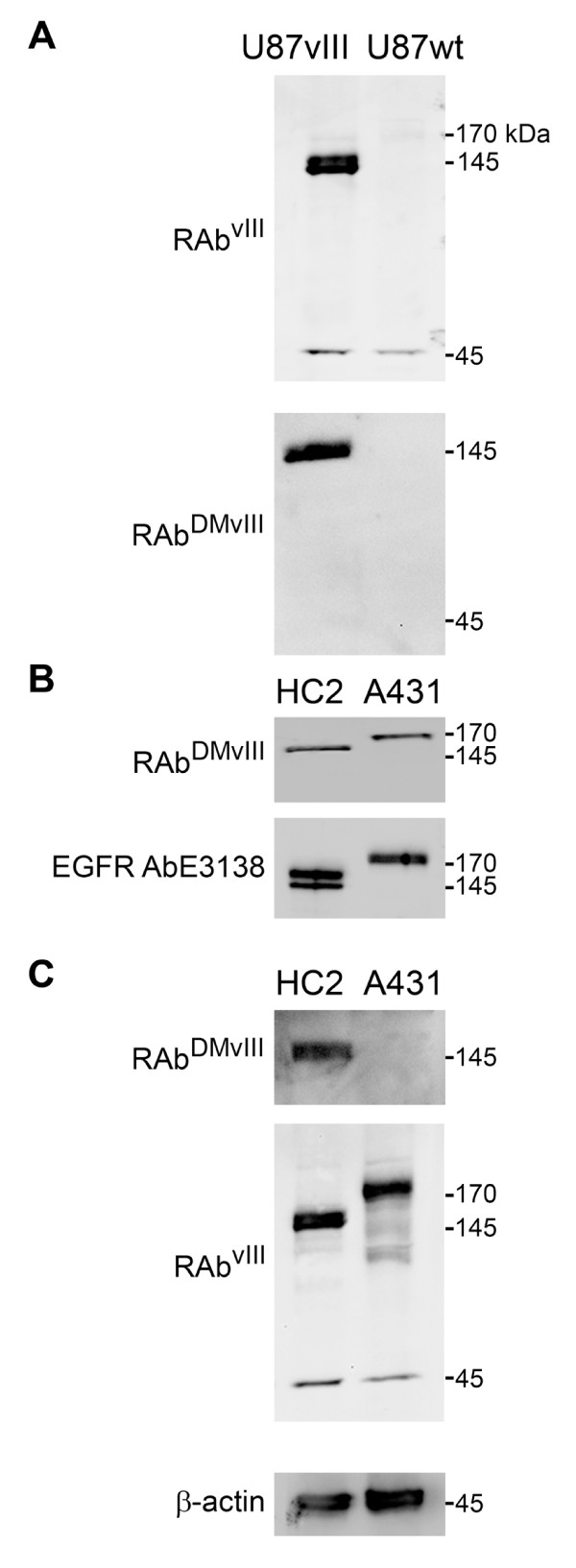
**Western Blot analysis of RAb^DMvIII ^antibody**. A) 30 ug of U87vIII and U87 wild type cell lysate loaded under reducing conditions on 4-20% SDS-PAGE. A) RAb^vIII ^recognizes 145 kDa band which corresponds to the EGFRvIII and 45 kDa unspecific protein band. A) RAb^DMvIII ^recognizes only 145 kDa protein band. B) RAb^DMvIII ^recognizes 170 KDa protein band in 30 ug of A431 cell lysate (cells overexpressing EGFR) and 145 kDa band in 30 ug of HC2 cell lysate under reducing conditions. B) EGFR antibody recognizes equal amount of protein in both HC2 and A431 cell lysate under reducing conditions. C) RAb^DMvIII ^under non reducing conditions detect only 145 kDa protein band whereas RAb^vIII ^still recognizes 145 kDa and 170 kDa protein bands in non reducing conditions. C) Actin antibody under reducing condition shows equal amount of protein loaded in the wells.

We constructed the first recombinant EGFRvIII antibody, RAb^vIII^, using the sequence of MR1-1, an scFv developed against EGFRvIII (Figure 1) [29]. To determine the specificity of RAb^vIII ^antibody for EGFRvIII, we used it for western blot analysis. Consistent with previously published data, the RAb^vIII ^antibody recognizes the 145 kDa EGFRvIII protein band in U87-vIII cells stably expressing EGFRvIII (U87-vIII) compared to the U87-MG untransfected cells (Figure 2A) [30]. The RAb^vIII ^antibody also faintly recognized the endogenous 170 kDa wild type EGF receptor and it strongly recognize a 45 kDa protein band in both U87-vIII and U87-MG control cells. We speculated that the 45 kDa protein was cross-reaction with another EGF receptor variant that we have recently identified, called mLEEK. MiniLEEK (mLEEK) is the result of the fusion of exon 1 to exon 23 and is predicted to be 45 kDa in size (Piccione et al., submitted). This also creates a glycine at the junction and the fusion junction (LEEKKGVTVWEL) bears some resemblance to the EGFRvIII sequence.

The crystal structure of MR1-1 in complex with the EGFRvIII peptide has been done by Landry et al.[31] This shows the valine at P9 position of the EGFRvIII peptide forms a hydrogen bond with tyrosine at H59 and H105 on MR1-1. The mLEEK peptide has a valine at the identical position. Since RAb^vIII ^potentially cross reacted with mLEEK, to eliminate the non-specific 45 kDa band we mutated the tyrosines at H59 and H105 to phenylalanine.

To determine if the mutated RAb^DMvIII ^has improved specificity towards EGFRvIII, we conducted another western analysis. We found that RAb^DMvIII ^now specifically recognizes only the EGFRvIII 145 kDa protein band and not the EGFR variant 45 kDa band (Figure 2A). We also more rigorously tested cross-reaction with wt EGF receptor by performing Western blots using lysates from A431 cells, which express 1 × 10^6 ^receptors per cell. When we studied the specificity of RAb^DMvIII ^towards the overexpressed wild type EGFR present in A431 cells we discovered that the RAb^DMvIII ^antibody did cross-react with the 170 kDa wild type protein (Figure 2B).

Consequently, to further study the specificity of the RAb^DMvIII ^under these conditions we expanded our analysis to include HC2 cells which expresses EGFRvIII at a level comparable to that of EGFR expressed by A431 (Figure 2B). Western analysis using anti-EGFR antibody E3138 from Sigma shows that there are similar amounts of wild type EGFR and EGFRvIII protein expressed in A431 and HC2 cells (Figure 2B).

Based on the study done by Johns et al. which showed that monoclonal antibody 806 recognizes overexpressed EGFR in A431 cells only under reducing conditions, we further analyzed both HC2 and A431 cell lysates under non-reducing western conditions [32]. Western analysis under these conditions shows that recombinant RAb^DMvIII ^recognizes only the EGFRvIII 145 kDa band and not the wild type EGFR 170 kDa band (Figure 2C) suggesting that under reducing conditions the EGFR epitope is somewhat more exposed and accessible to RAb^DMvIII ^[32]. On the other hand, the non mutated RAb^vIII ^consistently recognizes the 170 kDa wild type EGFR band even under non-reducing western conditions clearly illustrating that RAb^DMvIII ^is a more specific EGFRvIII antibody than RAb^vIII^.

### Antibody Affinity

**Figure 3:**
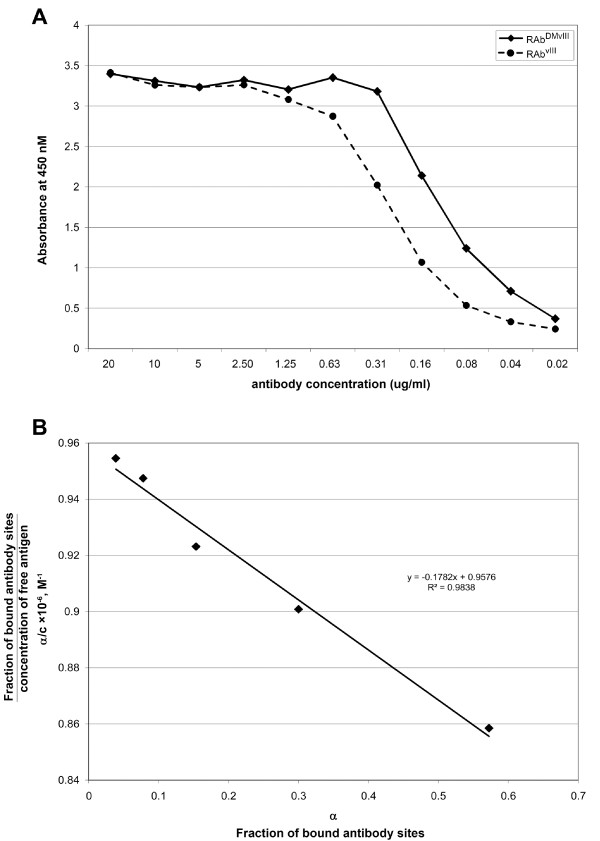
**Affinity determination of RAb^DMvIII ^by Saturation ELISA**. Calibration curve of the binding of RAb^DMvIII ^and RAb^vIII ^recombinant antibodies to coated EGFRvIII in the ELISA. A) The concentration range for RAb^DMvIII ^and RAb^vIII ^was 20 to 0.02 ug/ml (x axis) and the absorbance was read at 450 nm (Y axis). The assay was analyzed twice. B) Saturation ELISA shows the Scatchard plot of the binding of RAb^DMvIII ^to EGFRvIII. The resulting data is plotted as ratio of α/c (y axis) as function of alpha (x axis) where α is the fraction of bound antibody sites and c is the concentration of free antigen. The slope is -K which is the affinity constant of antibody and that is 1.7 × 10^7 ^liters/mole (M^-1^).

To determine the affinity constant of the antibody, we used the method described by Friguet et al. (1984) [33]. It is necessary that the concentration of the antibody used in the ELISA should be equal to or less than the value of dissociation constant of the antibody. Thus a preliminary ELISA was performed to deduce the dissociation constant of the antibody. From the preliminary ELISA, the dissociation constant of RAb^vIII ^and RAb^DMvIII ^is calculated to be approximately 1.7 μM and 886 nM, respectively, against EGFRvIII peptide (Figure 3A). This result suggests that RAb^DMvIII ^did not lose its affinity after mutation as RAb^DMvIII ^has a lower dissociation constant than RAb^vIII^.

The indirect ELISA was performed with RAb^DMvIII ^using a concentration of 117 nM, which is lower than its dissociation constant value of 886 nM. The OD values from this ELISA were placed in a Scatchard plot equation where the fraction of bound antibody sites α (determined from the OD values) is plotted on the X axis and the ratio of the fraction of bound antibody sites α to free antigen sites c, (α/c) is plotted on the Y axis. The slope gives the value of -K, the affinity constant of the antibody, which is 1.7 × 10^7 ^M^-1 ^(Figure 3B).

### Immunoprecipitation

**Figure 4:**
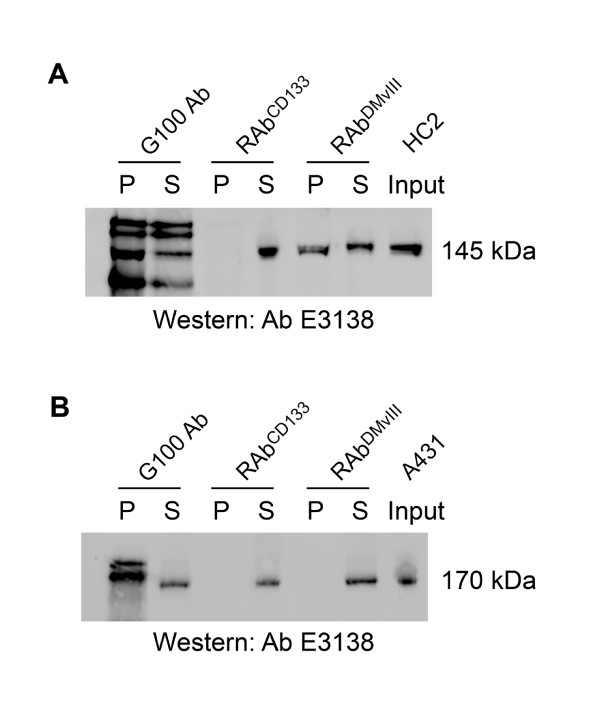
**Immunoprecipitation of EGFRvIII by RAb^DMvIII^**. Immunoprecipitation was performed with RAb^DMvIII^, Monoclonal EGFRvIII antibody and RAb^CD133 ^(negative control for our recombinant antibody) on HC2 and A431 cell lysates. A) HC2 lysates immunoprecipitated by Monoclonal EGFRvIII antibody, RAb^DMvIII ^showed 145 kda band corresponding to EGFRvIII in the pellet whereas 145 kda band is seen in the sup of RAb^CD133 ^when immunoblotted with E3138 antibody from sigma. B) On the other hand A431 cell lysate showed 170 kda protein band corresponding to EGFR in the supernatant of Monoclonal EGFRvIII antibody, RAb^DMvII1 ^and RAb^CD133^samples.

Immunoprecipitation of HC2 and A431 cell lysates was performed using RAb^DMvIII ^and G100 an anti-EGFRvIII monoclonal antibody (no longer commercially available). As a negative recombinant antibody control for EGFR wt and EGFRvIII, we also used RAb^CD133^, which utilizes the same expression vector as RAb^DMvIII ^but is directed against CD133 and recognizes neither EGFR wt nor EGFRvIII. HC2 cell lysates immunoprecipitated with RAb^DMvIII ^and G100 antibody and then further immunoblotted with E3138 antibody showed the expected 145 kDa band corresponding to EGFRvIII in the pellet whereas RAb^CD133 ^did not show the 145 kDa band in the pellet as it does not bind to EGFRvIII (Figure 4A) On the contrary, when A431 cell lysates were immunoprecipitated with either RAb^DMvIII^, G100 or RAb^CD133 ^and then immunoblotted with the E3138 antibody, the170 kDa bands corresponding to wt EGFR was found in the supernatant (Figure 4B). This suggested that even when abundant amounts of wt EGFR protein is present RAb^DMvIII ^did not bind to any wild type EGFR.

### Immunofluorescence

**Figure 5:**
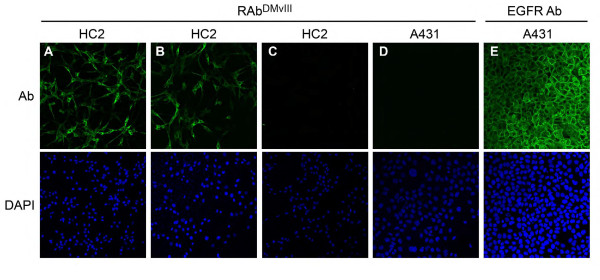
**Detection of specificity of RAb^DMvIII ^by Immunofluorescence**. A) Immunofluorescence study shows that RAb^DMvIII ^detects EGFRvIII protein in HC2 cells, B) Scrambled peptide competition shows detection of the EGFRvIII signal in HC2 cells by RAb^DMvIII^, C) Specific peptide (EGFRvIII) competition dampens the signal in HC2 cells, D) No signal is detected in A431 cells by RAb^DMvIII^, E) EGFR signal is detected in A431 cells by anti-EGFR antibody (sc-130, Santa Cruz).

Since the specificity of RAb^DMvIII ^is greatly enhanced under native conditions, we decided to test the recombinant antibody specificity under in vivo conditions. To this end, indirect immunofluorescence was performed on HC2 and A431 cells with RAb^DMvIII ^at a concentration of 0.25 μg/μl. Images were taken using a Leica SP2 AOBS confocal microscope. Consistent with previous findings, the RAb^DMvIII ^shows that the EGFRvIII protein is both membrane bound and has an intracellular localization (Figure 5A) [1,6,34]. To verify the specificity of the RAb^DMvIII ^antibody we conducted a peptide competition using the EGFRvIII epitope (LEEKKGNYVVTDHC). The presence of the EGFRvIII epitope completely blocked RAb^DMvIII ^from interacting with the EGFRvIII found in the HC2 cells (Figure 5C), as compared to the scramble epitope control which showed no decrease in detection of EGFRvIII (Figure 5B). Additionally, the A431 cells that over express wild type EGFR did not demonstrate any signal with RAb^DMvIII ^(Figure 5D), whereas the anti-EGFR antibody recognizes a strong EGFR wild type signal demonstrating that the A431cells retain high expression of the wild type protein (Figure 5E). Together these findings indicate again that this recombinant antibody is highly specific towards EGFRvIII.

### Immunohistochemistry

**Figure 6:**
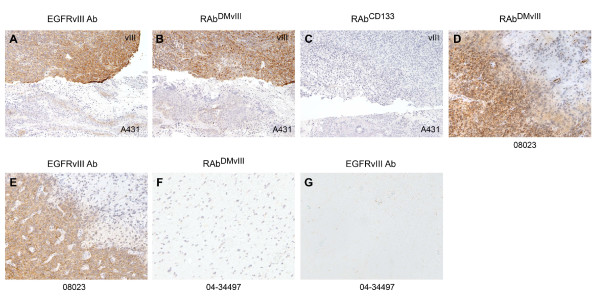
**Detection of EGFRvIII by RAb^DMvIII ^Immunohistochemistry**. (A-C) Immunohistochemical staining of U87vIII & A431 mouse tumor xenografts and (D-G) Glioblastoma section. A) Staining of A431 and U87vIII with polyclonal EGFRvIII antibody. B) RAb^DMvIII ^antibody staining, C) RAb^CD133^antibody staining. D) Glioblastoma 08-023 staining with RAb^DMvIII^. E) Glioblastoma 08-023 staining with polyclonal EGFRvIII ab. F) Glioblastoma 04-34497 stained with polyclonal EGFRvIII antibody and G) RAb^DMvIII ^staining of 04-34497.

Immunohistochemistry (IHC) is another sensitive biochemical test to monitor for the expression of EGFRvIII. To determine if the RAb^DMvIII ^antibody could be used for IHC, we used mouse tumor xenografts from HC2 and A431 cells (Figure 6A-C) and GBM cases (Figure 6D-G). The tumor xenografts contain both HC2 tissue (top) and A431 tissue (bottom). RAb^DMvIII ^shows strong positivity to the HC2 section which is comparable in intensity to slides stained with the positive control anti-EGFRvIII polyclonal antibody (Figure 6A &6B). In addition, the RAb^DMvIII ^antibody did not stain the A431 tissue sections (Figure 6A). The recombinant antibody RAb^CD133 ^was used as a negative antibody control (Figure 6C). We extended our findings to two GBM cases, one positive for EGFRvIII (08023) and one negative (04-34497). Consistent with the mouse tumor xenografts, the RAb^DMvIII ^antibody only stained the GBM positive for EGFRvIII and had no cross reactivity to the negative GBM case (Figure 6D &6F). As a control, we also stained these sections with affinity purified polyclonal anti-EGFRvIII and observed similar results (Figure 6E and 6G).

### Flow cytometry

**Figure 7:**
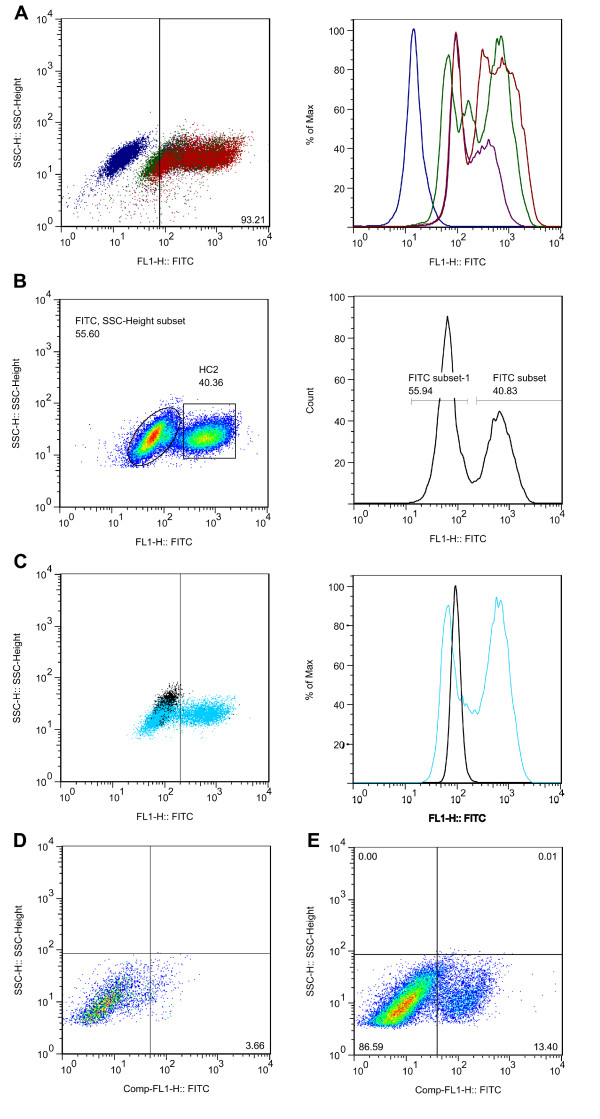
**Specificity of RAb^DMvII1 ^by Flow cytometry**. Flow cytometric analysis of surface expressed EGFRvIII. A) 1 × 10^6 ^cells HC2 cells were stained with increasing concentration of RAb^DMvIII^: 0.1 μg (purple), 1 μg (red) and 2 μg (green). The dot blot (left panel) and histogram in blue represents human-IgG1 isotype control. B) NIH3T3 and HC2 cells were mixed in a 1:1 ratio prior to staining with RAb^DMvIII ^revealing distinct identification of an EGFRvIII cell population. C) Overlay of flow cytometric plots of A431 (black) cells and HC2 cells (blue) stained with RAb^DMvIII^, showing clear selectivity for EGFRvIII expression over EGFR expression. D) Freshly dissociated human GBM tumor stained with RAb^DMvIII ^shows the presence of surface EGFRvIII on 13% of cells (right panel). The dot blot (left panel) represents human-IgG1 isotype control.

Because RAb^DMvIII ^efficiently detects surface EGFRvIII, we asked if it might be an effective reagent for flow cytometry experiments. Under native conditions we observed that RAb^DMvIII ^binds to surface expressed EGFRvIII very efficiently. Figure 7A shows an overlay of plots from HC2 cells stained with increasing concentration of RAb^DMvIII ^(0.1 μg, 1 μg and 2 μg per 10^6 ^cells). The dot blot and histogram in blue represents human-IgG1 isotype control and as low as 0.1 ug of RAb^DMvIII ^shows a significant shift in fluorescence intensity. Higher concentration of RAb^DMvIII ^(1 μg and 2 μg) show the presence of multiple peaks consistent with the immunofluorescence assay (Figure 5) which shows different levels of EGFRvIII expression. To look at the sensitivity of RAb^DMvIII ^in identifying and isolating EGFRvIII expressing cells, we mixed NIH3T3 and HC2 cells at a 1:1 stoichiometry prior to staining with RAb^DMvIII^. RAb^DMvIII ^was able to clearly identify and separate the HC2 cells from NIH3T3 cells.

One of the earlier concerns in developing the recombinant EGFRvIII antibodies was the potential cross-reactivity towards wt EGFR under conditions where the receptor is over-expressed such as that in A431 cells. Flow cytometry allows quantitation of any potential cross-reaction under native conditions. Figure 7C is the overlap of plots of A431 (black) and HC2 (blue) cells with RAb^DMvIII^. There is very little reactivity of RAb^DMvIII ^with A431 as compared to HC2 cells demonstrating that this reagent does not cross-react with wt EGFR. We further tested RAb^DMvIII ^for surface staining of freshly primary tumor cells and observed about 14% of cells to be EGFRvIII positive over the isotype control.

## Discussion

In this work, we have constructed a novel recombinant antibody, RAb^DMvIII^, which specifically recognizes EGFRvIII. Based upon a previously described scFv against EGFRvIII, we have performed further mutagenesis to eliminate cross reaction with a 50 kDa band. We have demonstrated the specificity of this antibody for EGFRvIII and not wt EGFR or any other protein by several methods. Unlike other monoclonal anti-EGFRvIII antibodies, this antibody is broadly useful because it recognizes the EGFRvIII epitope in several assays including ELISA, IF, IHC, western blot analysis, and flow cytometry.

A distinct advantage is that recombinantly produced antibodies require no animal for production but otherwise have the properties of conventional antibodies but with additional, improved properties. Indeed, the RAb^DMvIII ^has been engineered to have an IgG Fc region, which confers long serum half life, and has selected to support secondary immune functions, such as antibody dependent cellular cytotoxicity (ADCC) and complement mediated cytotoxicity (CMC) [35,36]. Studies have demonstrated that Fc-containing BsAb and other fusion proteins do retain the full effector mechanisms of the Fc component [36,37].

Effective tumor targeting requires that the antibody should have an affinity for its tumor antigen but extremely high affinity may in fact be detrimental [38]. Previous studies have shown that very high affinity interactions of 10^9 ^M^-1 ^between antibodies and tumor antigens may actually impair efficient tumor penetration of the monoclonal antibodies and thus diminish effective in vivo targeting [39]. Fujimori et al. [40] and Van Osdol et al. [41] have also demonstrated that higher affinity monoclonal antibodies do not homogenously distribute and show less killing of the target cancer cells. According to Robinson et al., a high antibody affinity restricts the localization and tumor penetration of Fv antibody molecules [42]. In another study, Langmuir et al. [26] have shown that a lower affinity mAb of 5.2 × 10 ^7 ^M^-1 ^was nearly as toxic as a mAb with an affinity of 1.9 × 10^9 ^M^-1^. Our antibody has an affinity of 1.7 × 10^7 ^M^-1 ^as shown by saturation ELISA so it could be a reasonable candidate for therapeutic studies.

In separate studies, we have already begun to explore the effectiveness of the RAb^DMvIII ^in targeting tumor spheres derived from glioblastoma tumors. These experiments have already shown that this reagent specifically lyse the cells expressing EGFRvIII and spare normal neural stem cells (Mitra et al., submitted). We performed cytotoxicity assays using a coupled luminescence method on various EGFRvIII transfected cell lines with human NK cells as effectors and found that this reagent significantly induces cell killing of EGFRvIII transfected cells. RAb ^DMvIII ^also remarkably reduces the sphere initiating frequency on GBM cells but not in normal cells as it has been shown that EGFRvIII is required for the maintenance of glioma growth in vivo [43]. To further investigate this reagent in mouse models, two GBM sphere lines were incubated with either RAb^DMvIII^, RAb^CD133 ^or a non-specific human control antibody and orthotopically injected into the cortex of NOD-SCID mice. Tumor formation was analyzed after 26 weeks which revealed a higher incidence of human tumor cells engraftment with RAb^CD133 ^and Hu-IgG1 as compared to RAb^DMvIII ^(Mitra et al., submitted). In order to provide this reagent to other researchers and for future studies, we are planning to scale up antibody production using hollow fiber bioreactor system. Hence this antibody could be of great significance in the field of brain tumor research.

## Conclusion

Despite significant advances in the field of neuro-oncology and immunology, there is no widely available EGFRvIII antibody for scientific or clinical use. Furthermore, there is no EGFRvIII antibody clinically proven to be used on patients because of cross reactions and other high risk allergic reactions due to murine Fc portion of the monoclonal antibodies. RAb^DMvIII ^has shown high specificity towards EGFRvIII and thus holds great potential to be further investigated for therapeutic purposes.

## Methods

### Cell Culture

U87-MG, HEK293, A431, and HC2 cell lines were obtained from the American Type Culture Collection (ATCC). U87-MGvIII, a human glioma cell line stably transfected with EGFRvIII, was generously donated by Dr Donald O'Rourke. Cell lines were grown in Dulbecco's modified Eagle's medium (DMEM) (glucose at 4.5 g/L) supplemented with either 10% fetal bovine serum or calf serum as per cell line requirement, 100 units/ml penicillin, 100 μg/ml streptomycin, and 100 μg/ml kanamycin sulfate. The U87-MGvIII cell lines were maintained in Geneticin (G418) selection at 500 μg/mL.

### Tumor Sections for Immunohistochemistry

NOD-SCID mice were injected subcutaneously with either 1 × 10^6 ^U87-MGvIII or A431 cells in the right hind flank of athymic mice. After fourteen days, the mice were sacrificed and the flank tumors were dissected out and fixed in 10% formalin. Four micron tissue sections of both A431 and U87-MGvIII tumors were cut and mounted on the same slide. Cut and mounted primary human glioblastoma tumor sections were obtained from Stanford University brain bank under IRB approved protocols.

### Plasmid Construction and Cloning

The pBudCE4:Her2/neu:CD16 bispecific minibody was a kind gift from Dr. Louis Weiner [44]. This construct has an IgG1 CH3 constant domain linked to one single chain variable fragment (scFv) against Her2/neu and one scFv against CD16 in a di-body format on the pBudCE4 vector backbone. The Her2/neu and CD16 variable fragments were replaced with MR1-1 [21] as described below. MR1-1 is a scFv against EGFRvIII selected from a phage display library which was derived from MR1 (scFv), an antibody Fv fragment that recognizes an epitope within the EGFRvIII-specific sequence. The MR1-1 scFv was artificially synthesized based on sequence available from Genbank (accession no. U76382).

The plasmid construct pBudCE4:Her2/neu: CD16 was digested with Not I and Bgl II to remove the Her2/neu scFv. The artificially synthesized MR1-1 was amplified using primers AAGGAAAAAAGCGGCCGCACCATGGAGACAGACACACTCCTGCTATGGGTACTGCTGCTCTGGGTTCCA

GGTTCCACTGGTGGTGACGACTACAAGGACGACGATGACAAGCAGGTGAAACTGCAGCAGTCT (scFvEGFRvIII-Not1-ATG/Igk (F)) and GGAAGATCTACCGCCACTGCCACCTCCGCCTGAACCGCCTCCACCACTCGAGCCACCTTTGATTTCCAGCTTGGTGC (scFvEGFRvIII-Linker/BglII(R)) and ligated into the scFv Her2/neu position. The resulting recombinant antibody was then digested with Sfil and Xba1 to remove the scFv for CD16. Primers ACATTGGTGCCGAAACCTATTCACTGCTTACTCGCGGCCCAGCCGGCCCAGGTGAAACTGCAGCAGTCT (scFvLEEK-Sfil(F)) and GCTCTAGAACCGCCACTGCCACCTCCGCCTGAACCGCCTCCACCACTCGAGCCACCTTTGATTTCCAGCTTGGTGC (scFvLEEK-XbI/linker(R)) were used to amplify the second EGFRvIII scFv and ligated into the scFv CD16 position. The plasmid pBudCE4(scfvEGFRvIII) has the CH3 gene with "knobs-into-holes" configuration [45]. Additionally the V5 epitope and His6 tag sequences are cloned at the 3'-end of one anti-EGFRvIII binding arm, whereas the MYC epitope and His6 tag sequences were cloned at the 3'-end of another anti-EGFRvIII binding arm (Figure 1). A construct with two scFvs against CD133 was created in a similar fashion as a control.

### Site Directed Mutagenesis

Site directed mutagenesis was used to change tyrosine H59 of the CDRH2 domain and tyrosine H105 of the CDRH3 domain to phenylalanine for both the anti EGFRvIII scFv sequence inserts (200519-5, Stratagene). Primer CTATTCTCCTTACTCTTTTGCTATGGACTACTGGG (F) and primer CCCAGTAGTCCATAGCAAAAGAGTAAGGAGAATAG (R) were used for the H105 mutation while primer ACTGGCGGTTATAATACCTTCTATTCAGACAATGTAAAG (F) and primer CTTTACATTGTCTGAATAGAAGGTATTATAACCGCCAGT (R) were used for the H59 mutation. The resulting mutated vector is called pBudCE4(DMscfvEGFRvIII) and the antibody produced by this construct is called RAb^DMvIII ^whereas the antibody produced by pBudCE4(scfvEGFRvIII) and pBudCE4(scfvCD133) are called RAb^vIII ^and RAb^CD133 ^respectively.

### Transfection, Collection and Purification of Antibody

The pBudCE4scfvEGFRvIII, pBudCE4scfvCD133 and pBudCE4DMscfvEGFRvIII plasmids were stably transfected into HEK293 cells using calcium phosphate, and the positive clones were selected using zeocin at 400 μg/ml. Media was collected from the stably transfected cells and the His-tagged antibodies were isolated and purified using Ni-NTA agarose (R901-15, Invitrogen) according to the manufacturer's recommended protocol. The antibodies were eluted using 500 mM imidazole. The fractions containing the antibodies were identified through Coomassie staining of SDS PAGE. The fractions containing the antibody protein were combined and dialyzed against phosphate buffer solution (PBS). The protein concentration was determined by a Bio-Rad Dc protein assay.

### Western Blot Analysis

Confluent HC2 and A431 cells were washed with ice-cold PBS and lysed using a buffer containing 10 mM Na_2_HPO_4_, 150 mM NaCl, 1% Triton X-100, 0.5% sodium deoxycholate, 0.1% sodium dodecyl sulfate, 0.2% sodium azide, 0.004% sodium fluoride, 1 mM NaVO_4_, 25 mM β-glycerophosphoric acid, 100 μg of phenylmethanesulfonyl fluoride/ml, 10 μg of aprotinin/ml, and 10 μg of leupeptin/ml (pH 7.35). Cell lysates were centrifuged at 12,000 × *g *for 10 min at 4°C to clear out cell debris, and protein concentration was determined by Bio-Rad DC protein assay. For gel electrophoresis under non-reducing conditions, forty micrograms of cell lysate was prepared in 6x Laemmli Sample Buffer lacking β-mercaptoethanol and the samples were not boiled. The proteins were resolved on 4-20% Tris-glycine SDS-PAGE gels (Invitrogen) and transferred to nitrocellulose membrane for immunoblot analysis. The membranes were blocked with blocking buffer 5% Blotto (100 mM Tris [pH 7.5], 0.9% NaCl, 0.1% Tween 20 with 5% nonfat dry milk) and incubated with the following antibodies: RAb^vIII^, RAb^DMvIII^, mouse anti-actin (MAB1501R, Chemicon), and mouse anti-EGFR (E3138, Sigma Aldrich). Horse radish peroxidase (HRP) affinity pure Donkey anti-Human IgG (709-035-149, Jackson Immunoresearch) was used as the secondary for the recombinant antibodies. Proteins were detected with ECL reagents (#RPN2106V1 and #RPN2106V2, GE Health care).

### Immunoprecipitation

HC2 (EGFRvIII transfected) and A431 (EGFR wt transfected) cell lysates were isolated as described in the western blot section and 300 μl of lysate was incubated with 12 μg of G100 antibody (formerly commercially available from Zymed lab, now discontinued), RAb^CD133 ^and RAb^DMvIII ^for 1 hr at 4°C. Forty μls of washed 50% slurry of protein Gammabind G Sepharose (17-0885-01, GE Healthcare) was added to each lysate/antibody mix and incubated overnight at 4C. Immunoprecipitates were washed three times with the same lysis buffer and resuspended in sample buffer containing β-mercaptoethanol. Immunocomplexes were resolved on 4-20% Tris-glycine SDS-PAGE gels under normal reducing conditions and transferred to nitrocellulose membranes for immunoblot analysis by mouse anti-EGFR antibody (E3138, Sigma Aldrich).

### Immunofluorescence

HC2 and A431 cells were fixed in 4% paraformaldehyde, permeabilized with 0.5% Triton X, and blocked with 5% goat serum (0060-01, Southern Biotech). The following antibodies were used for IF: RAb^DMvIII^, mouse anti-EGFR (EGFR (528): sc-120, Santa Cruz Biotechnology). FITC goat anti-human (A11013, Jackson Immunoresearch) and FITC goat anti-mouse (A11029, Jackson Immunoresearch) were used as secondary antibodies. Images were obtained using a Leica SP2 AOBS Confocal Laser Scanning Microscope. For peptide competition, ~20 times higher molar concentration of EGFRvIII peptide (LEEKKGNYVVTDH-C, Genscript) and scrambled peptide (WELKVNGTKDEYH-C, Genscript) was incubated with the primary antibody at 37°C for 1 hour and the mixture was centrifuged at 14000 × g for 5 min. The supernatant was used at the primary antibody incubation step of immunofluorescence protocol.

### Immunohistochemistry

Slides were deparaffinized in xylene, and rehydrated in decreasing percentages of ethanol. 3% hydrogen peroxide was used to block endogenous peroxidase and the slides were placed in Diva Decloaker (BioCare Medical) and microwaved for 13 minutes 10 seconds for antigen retrieval. After a cooling down period, the slides were treated with Sniper Universal serum (BioCare Medical) and then incubated with the following antibodies at 0.5 μg/ml overnight at 4°C: Rabbit anti-EGFR antibody, RAb^CD133 ^(recombinant negative control) and RAb^DMvIII^. Each slide was then incubated with the appropriate secondary and/or tertiary antibody. The secondary antibodies used were: HRP-Anti Rabbit (BioCare Medical), anti-Human IgG rabbit (I9135, Sigma), HRP anti-rabbit antibody (BioCare Medical). Protein expression was detected by betazoid DAB (BioCare Medical). The slides were also counterstained with hematoxylin. Glioblastoma slides were analyzed in a similar fashion to the A431 and U87-MGvIII slides except that the slides for RAb^DMvIII ^were blocked with equal volume of background sniper (BioCare Medical), 5% Blotto and normal human serum (31876, Fisher) and the secondary antibody for RAb^DMvIII ^was rabbit anti-V5 (A00623, Genscript) followed by the tertiary antibody, which was HRP-anti-rabbit (BioCare medical).

### Enzyme-Linked Immunosorbent Assay (ELISA)

To determine the affinity constant of the RAb^DMvIII ^a preliminary ELISA was performed to find the concentration of the antibody. This preliminary ELISA was performed with both RAb^DMvIII ^and RAb^vIII ^antibodies to compare the dissociation constant of both the antibodies. A dilution series of antibodies RAb^DMvIII ^and RAb^vIII ^(20 μg/ml-0.01952 μg/ml) in 5% blotto was used. ELISA plates were first coated with 10 μg/ml of EGFRvIII peptide in coating solution in duplicate for each antibody overnight at 4C. The next day the plates were washed with 1× wash buffer (50-63-00, KpL) and 5% blotto was added for 1 hr at RT. Thereafter the respective plates were incubated with different dilutions of RAb^DMvIII ^and RAb^vIII ^ranging from 20 μg/ml to 0.02 μg/ml in 5% blotto for 1 hr at RT. The plates were washed 3 × 10 min each with 1× wash buffer, and then affinity pure Donkey anti-Human IgG (709-035-149, Jackson Immunoresearch) was applied at a 1: 5000 dilution in 5% Blotto for 1 hr at RT, and then the plates were washed 3 × 10 min each. 100 μl of 1× developing reagent was added to each reaction well, and after 10 min., 100 μl of stop solution 1N HCl was added and the absorbance was read at 450 nm. All curves reached a maximum value, which indicated that the reactions proceeded until saturation. The data was plotted as the absorbance at 450 nm versus different antibody concentrations. This gave an equilibrium dissociation constant, K_d_, for RAb^DMvIII ^of 886 nM and for RAb^vIII ^of 1.7 μM.

### Determination of Affinity Constant

Indirect ELISA was performed to determine the affinity constant of the recombinant antibody. This method developed by Friguet et al [33] uses a solid-phase assay for measuring the amount of free antibody present at equilibrium in an antigen-antibody reaction mixture. Since the RAb^DMvIII ^has a lower dissociation constant than RAb^vIII ^as shown by preliminary ELISA we decided to perform indirect ELISA with RAb^DMvIII ^only.

Two ninety-six well ELISA plates (353279, BD Falcon) were coated overnight at 4°C with EGFRvIII peptide LEEKKGNYVVTDH-C at a decreasing concentration from 40 ug/ml to 2.5 μg/ml in coating solution (50-84-00, KpL). Wells were then blocked with 5% nonfat dry milk for 1 hr at room temperature. In parallel tubes, antibody at 234 nM is incubated with double the concentration of antigen used in the plates that is (80 μg/ml-5 μg/ml) in equal volume for final concentration of antibody to be 117 nM (less than the dissociation constant of RAb^DMvIII ^) and final antigen concentration of (40 μg/ml-2.5 μg/ml) for 30 min at RT, and then the antibody-antigen mixture is transferred to the wells of the first antigen coated microtiter plate and incubated for 15 min. After this incubation, the contents are then transferred to the second antigen coated plate and incubated for another 15 min. The plates were washed with wash buffer (50-63-00, KpL) and secondary donkey anti-Human antibody from Jackson ImmunoResearch was added for 1 hr incubation at RT. After further washes of 3 × 10 min each, 100 μl of substrate (53-00-02, KpL) is applied and 100 μl of stop solution (1 N HCl) is added after 10 min. The absorbance was read at 450 nm in an ELISA plate reader. The data is plotted as the fraction of bound antibody sites versus the ratio of (fraction of bound antibody/concentration of free antigen). The slope gives the value of K, which is the affinity constant.

### Flowcytometry

Flowcytometric analysis was performed using RAb^DMvIII ^for native cell surface expressed EGFRvIII. U87-MGwt, U87-MGvIII, A431, and HC2 cell lines were dissociated using TryplE^® ^(Invitrogen, Carlsbad, CA). Cells were resuspended in flow cytometry buffer, consisting of 1× HBSS, pH 7.2, containing 1.55 g/l glucose and 0.1% fraction V of bovine serum albumin (BSA; Sigma-Aldrich, St. Louis,). Cells were counted and diluted to a density of 10^6 ^cells per milliliter of buffer. 1 μg of RAb^DMvIII ^was used per 10^6 ^cells unless otherwise specified. Cells were analyzed on a BD LSR-I flowcytometer (BD Biosciences). Unlabeled cells and cells labeled with isotype control (human IgG1) were used to set gating parameters between positive and negative cell populations. Cell aggregates and small debris were excluded from analysis or isolation on the basis of side scatter (measuring cell granularity) and forward scatter (measuring cell size); dead cells were excluded from analysis on the basis of viability dye fluorescence (propidium Iodide). Fluorescent intensities for cells in the population were point-plotted on two-axis graphs or histogram using FlowJo software (Tree Star Inc.).

## Authors' contributions

PG performed the majority of the experiments, analyzed the data and wrote the manuscript, SYH constructed the primers for scFv EGFRvIII amplification, MHM designed the conditions to perform PCR for scFv EGFRvIII amplification, SSM performed and analyzed FACS, GL & RTN have been involved in the editing, revising, and formatting of the manuscript. AJW initiated the study, analyzed the data, supervised the overall project and wrote the manuscript. All authors read and approved the final manuscript.
